# Do government outlays crowd-out private consumption? Evidence from the European Union

**DOI:** 10.1371/journal.pone.0336229

**Published:** 2026-01-02

**Authors:** Ömer Faruk Bölükbaşı, Olcay Çolak

**Affiliations:** 1 Department of Economics, Faculty of Economics and Administrative Sciences, Recep Tayyip Erdoğan University, Rize, Türkiye; 2 Department of Economics, Faculty of Economics and Administrative Sciences, Uşak University, Uşak, Türkiye; University of Salerno: Universita degli Studi di Salerno, ITALY

## Abstract

The relationship between private consumption expenditure and public expenditure represents a recurring theme in macroeconomics, with relevance to both empirical and theoretical discourse. However, there is a lack of consensus on its direction. Accordingly, this study aims to examine whether public expenditure rules out private consumption expenditure for European Union during the period of 1995–2022. The findings indicate that public expenditure has a positive effect on private consumption expenditure over the long term, thereby corroborating Keynesian theory. However, except the defense expenditure, the findings demonstrate the complementary effect of public expenditures on private consumption expenditures. Moreover, disposable income has a positive influence in all model specifications, which corroborates the Keynesian Absolute Income Hypothesis. Considering the findings, this study also suggests some policy recommendations for the future.

## Introduction

Results of fiscal policy on diverse economic aggregates present a compelling issue within the macroeconomics, with both theoretical and empirical aspects. As a substantial instrument, government outlays play important roles in affecting various macroeconomic indicators. In this context, the interaction between public outlays and private consumption has been one of the most challenging debates. Despite rising tendency of the empirical studies in recent period, this relationship has a significant theoretical ground and tradition in macroeconomics. Accordingly, theoretical debates have focused on the two main macroeconomic notions. In Keynesian view, rising government outlays may have both nominal and real effects depending on which the actual output is below the full employment level or not [1]. Since the representative non-Ricardian individual’s consumption depends on current disposable income, easy fiscal policy increasing government outlays would likely stimulate the aggregate demand, output, and thus income. When the economy is at underemployment level of output, fiscal stimulus would generate real impact so that the rising government outlay would magnify private consumption depending on the magnitude of the multiplier and hence marginal propensity to consume.

Extension of the traditional Keynesian view regarding on which public and private expenditures are complements were augmented by [2] under the New Keynesian paradigm in which households’ consumption is purely determined by their disposable income by assuming the validity of price stickiness and non-competitive labor market. Under the validity of non-competitive environment for labor market, households tend to supply their labor with respect to the wages that are determined by reconciliation of the economy-wide unions. In this setting, a positive shock in government spending tends to accelerate aggregate demand via traditional Keynesian multiplier mechanism to the extent that firms react this increase by demanding more labor. Under the monopolistic structure of the labor market, real wages tend to increase, which, in turn, translates into an uplift in consumption [3].

Contrary to the Keynesian view, the neoclassical tradition and its extensions argue that these two types of goods are substitutes rather than complements. As emphasized by the advocates of the Real Business Cycle (RBC) theory, an exogenous upsurge in government outlays tends to rule out private consumption. Therefore, fiscal policy becomes inefficient for stimulating aggregate demand and output. The fact that rising government outlays tend to displace private consumption is substantially linked with how the former is funded. In the case of rising taxes, substitution and wealth effects arise, and the former intercedes with the reaction of the households to the rising taxes by supplying more of labor force [4]. On the contrary, wealth effect intercedes in a negative direction with which shrinking disposal income due to rising taxes. Since disposable income, which is calculated by deducting taxes from personal income and plays an important role in determining private consumption, rising taxes would likely reduce private consumption as long as the decrease in disposable income [5]. The RBC theory argue that negative wealth effect outperforms the positive substitution effect and thus, private consumption tends to decline with rising taxes on personal income [4].

The crowding-out effect may also arise in cases of so-called *deficit financing*, in which governments issue and sell the bonds to finance the budget deficits [5]. Accordingly, governments are inclined to increase interest rates for the available bonds in the financial market to borrow in financing the budget deficits. Ultimately, lack of sufficient funds would likely elbow out private sector from the economic activities to the extent that shrinkage in private consumption and private investment expenditure may reduce the aggregate demand and output. Beyond taxes and deficit financing, government expenditure can be financed by printing money as well. In this case, inflationary pressures may arise, which may result in a subsequent increase in interest rates [6] and thus, rising interest rates would induce declining tendency in private consumption by depreciating the value of financial assets that households own. On the other hand, households’ reactions are important as long as how the government outlays are financed through the aforementioned ways. Known as the Ricardian equivalence hypothesis, households have optimal foresights so that a balanced budget policy, if increased public expenditure is equally financed regardless of the form of financing, would have no impact on private consumption [4]. Yet, households adjust their spending and saving behaviors to align with the constraints imposed by the government budget [4,7,8].

Besides the aforementioned theoretical discussions regarding the impact of general government outlays on private consumption, it is also necessary to mention the impact of various types of government outlays on private consumption, which has deep theoretical insights. The secret behind these theoretical and empirical discussions lies on the breakdown of the public spending into two broad categories, which are military and non-military or civilian expenditures. The theoretical discussions date back to the pioneering study carried out by [9] claimed that government expenditure would influence private consumption by generating welfare to the households. Hence, depending on the mechanism funding the government outlays may influence the consumption decision of the households [10].

[10] and [11] argued that the impact of government outlays on private consumption is notably depends on whether military spending is involved or not. [2] demonstrated that rising government outlays were inclined to increase private consumption when military spending was excluded [10]. In a bifurcated approach, the rationale behind the view that how military spending influences private consumption manifested by [11], who addressed the empirical assessments introduced by [12,13], and [14]. The first approach, which was empirically tested by [12] and [13], states that under fixed budget constraint, if government decides to increase military spending, then trade-off between military spending and private consumption emerges [11]. In order to finance the rising military spending, governments either tend to cut non-military expenditures (i.e., health, education), which may cover a significant proportion of the representative household’s budget, or decide to increase taxes. Ultimately, [12] and [13] asserted that private consumption reacts negatively to the rising military spending [11]. [14], who stated that rather than non-military spending, military expenditures might have heterogeneous effects on private consumption decisions, bases the second approach on the view. This situation applies especially to peace times, when governments decide to reduce taxes, which in turn could be regarded as tax refund to the consumers. The associated rise in disposable income might induce higher demand for goods and services [11].

In line with the aforementioned arguments, layout of the remainder is as follows. The next section will address the prevailing tendencies in private consumption and general government expenditure by asserting relevant figures for the EU countries. In the light of the aforementioned theoretical discussion, Section 3 thoroughly revamps the extant literature. Section 4 argues the data- and model-related issues, whereas Section 5 suggests the estimation methods. Accordingly, Section 6 reports empirical findings and is devoted to the discussion of those findings. Yet, the paper ends with policy recommendations in Section 7.

## Stylized facts

**Fig 1:**
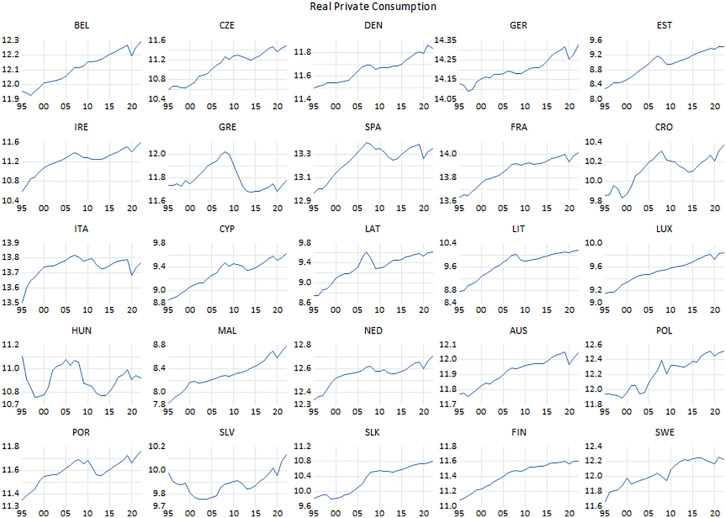
Real private consumption.

**Fig 2:**
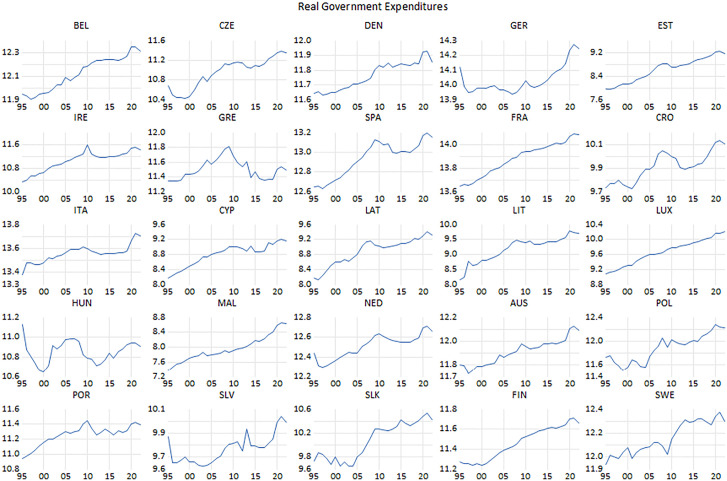
Real government expenditure.

It is necessary to assess whether the economy is heading towards an inflationary trend or whether it has stagnated. This is because the share of consumption expenditure in GDP may vary by the development level of economies. There are many variables that may affect private consumption expenditure. Additionally, changes in economic policy may also affect private consumption expenditure. Concurrently, fiscal policy and alterations in public expenditure, a pivotal instrument of fiscal policy, can impede expenditures of the private sector. as emphasized in the [15], the EU-wide household final consumption accounts for 52.1% of the GDP in 2023, with a slight increase compared to 2022, when household final consumption constituted 51.8% of the GDP. Moreover, the share of general government expenditure was 21.1% of the EU’s GDP in 2023. Accordingly, Figs 1 and 2 illustrate the general tendencies in private consumption and general government expenditure for the EU countries.

Notably, compared to the beginning of the sample period, private consumption expenditure has been on an upward trajectory over the last two decades. This persistence in consumption is particularly salient in the face of significant financial turbulence in 2008, the subsequent debt crisis, the onset of the pandemic, and the resultant economic downturn. Greece, which was most adversely affected by the financial turmoil in 2008 and the subsequent debt crisis, is strikingly distinctive from the other states in the sample. There also were fluctuations in consumption expenditures in Spain, Italy and Portugal, which were adversely influenced by the debt crisis, even though not as sharply as Greece. As for the CEE countries, the volatility in incomes and prices due to the measures aiming to stabilize the economies led to a fluctuation in private consumption in the second half of the 1990s in some countries. Most notably, Hungary stands out from other countries. Because of the austerity policies implemented to ensure price stability since the second half of the 90s, private consumption expenditures plummeted due to the contraction in demand. Slovenia and Poland are other CEE countries that faced with a similar situation. Meanwhile, the course of general public expenditure is presented in Fig 2.

Government outlays increased in all countries at the end of the period compared to the beginning. However, it should be noted that there are some exceptions to this general rule. Indeed, there are instances of cutbacks in public expenditure, which occurred in the context of austerity measures taken within the framework of the financial turmoil of 2008 and the debt crisis in Europe. These two crises had a severe impact on certain countries. While government expenditures in the EU-14 countries, which are constituent and former members of the Union, underwent an escalating tendency throughout the period examined in this study, Greece significantly stands out from the other countries. More precisely, as public expenditure has plunged dramatically a result of the austerity policies implemented to cope with the debt crisis. Notwithstanding, in the late 90s, the adoption of the common currency and the monetary convergence criteria designed within the framework of the Maastricht Criteria also influenced in the downward trend of public expenditure in some former members of the EU, besides the EU’s Stability and Growth Pact, which came into effect in 1997. The same partially applies to Belgium, the Netherlands, Austria, Spain, and Italy, as well as Germany. In addition, substantial proportion of the CEE countries made effort to reduce their budget deficits to align with the Maastricht Criteria, with the aim of stabilizing their economies in the accession process to the EU and Eurozone. Thus, this fact became one of the most important factors in reducing public outlays. It can be observed that government expenditure has recently been reduced within the framework of the tight fiscal policy, as well as the tight monetary policy, implemented by countries to combat the inflation problem that emerged with the gradual lifting of the lockdowns due to the COVID-19 pandemic.

**Fig 3:**
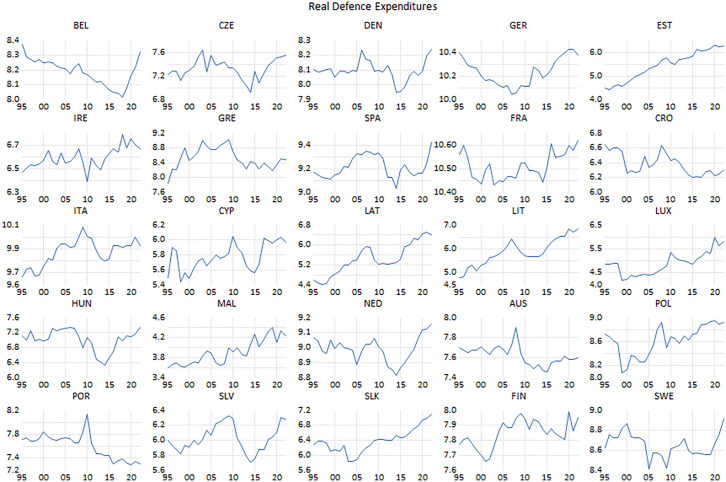
Real defense expenditure.

Meanwhile, public expenditure has also soared due to a number of circumstances, when its breakdown with respect to each component is considered. For instance, defense expenditure has surged in recent decade due to some compelling actions. In 2006, the NATO members committed to increasing their military spending up to 2% of their respective GDP. However, it wasn’t implemented until the Russian invasion of Crimea. After the Wales Summit of the NATO in 2014, the member states started to increase their military outlays as it was projected to reach 2% of GDP target due to the fear of possible Russian attack. As such, the struggle against illegal migration and yet, the recent warfare between Russia and Ukraine have enforced the majority of the member states to increase their military spending. In this respect, Fig 3 highlights the tendencies in defense expenditure by countries. With the exception of a few countries (i.e., Portugal), the member states are inclined to increase their military outlays in recent years.

**Fig 4:**
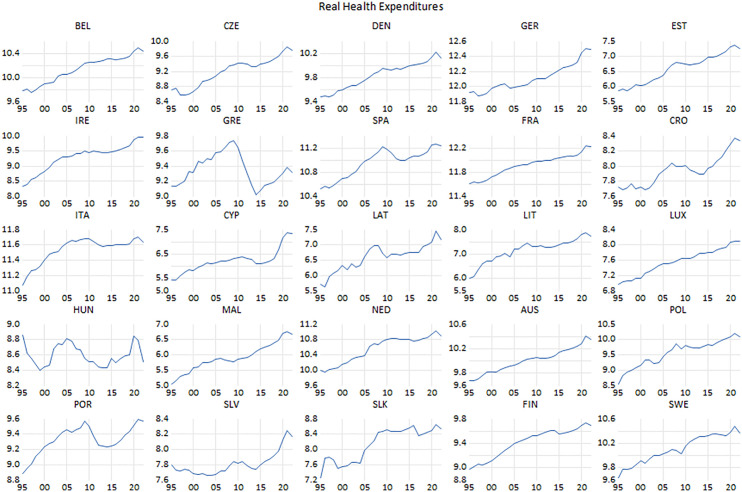
Real health expenditure.

**Fig 5:**
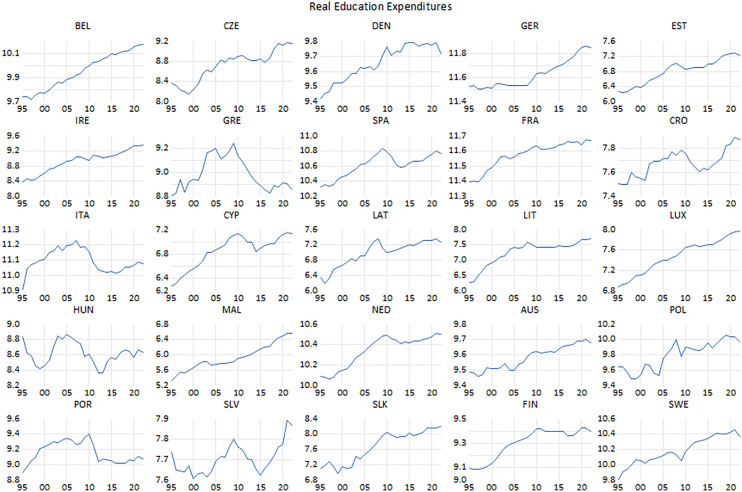
Real education expenditure.

Alongside defense expenditures, public spending on education and health accounts for a relatively large proportion of households’ budgets. Distinct from defense expenditures, these two expenditures are more productive in nature, given their potential contribution to human capital accumulation, and hence likely to contribute for economic growth. In this respect, Figs 4 and 5 illustrate the outlook for both types of expenditure.

Accordingly, both types of outlays have exhibited an upward trend to the extent that helping the general government expenditure to soar, barring the periods of the Eurozone crisis and the global financial crisis. Particularly, at the end of the sample period, the impact of the COVID-19 pandemic was effective in helping to increase general government outlays through health expenditures as part of the efforts to combat the pandemic. Nonetheless, the accompanying increase in education expenditures has been milder than health expenditures. Besides the global financial turmoil and Eurozone crisis, potential reasons that have adversely influenced expenditure could be the COVID-19 pandemic, security issues due to the conflict between Russia and Ukraine, and macroeconomic instabilities emerged by the soaring energy and food prices. Thus, the impact of these factors has manifested itself in different ways across countries, with some countries (i.e., Greece, Italy, and Portugal) experiencing a decline in education expenditures over time. On the contrary, in some Northern European countries (i.e., Sweden, Finland, and Denmark) and some former founding members (i.e., Austria, Germany, France, Belgium, the Netherlands, and Luxembourg) of the Union, the detrimental effects of these factors have been less pronounced, and education expenditures have remained at a high level. Furthermore, in some Central and Eastern European countries (i.e., Czechia, Croatia, Estonia, Latvia, Lithuania, Slovakia, and Poland) that joined the Union in 2004 and thereafter, education expenditures have increased despite the foregoing headwinds with the exception of Hungary and Slovenia, where education expenditures have exhibited relatively sporadic tendency in recent periods.

## Literature review

Even though the nuanced interaction between government outlays and private consumption has garnered significant interest among the scholars, there is no uniform tendency between these two main components of aggregate demand in the empirical literature so far. Therefore, the empirical studies provided intricate results. The results of empirical studies tend to support either the Keynesian view or the neoclassical approach, depending on the methodology, sample selection and variables that proxy government purchases and private consumption. The Keynesian view argues the validity of the crowding-in effect, while the neoclassical approach, particularly the RBC theory, emphasizes the validity of the crowding-out effect. Moreover, the notion of substitutability and/or complementary nexus more or less pertinent to the structural properties of the selected economies as to whether composition of government outlays and development levels vary across the countries and regions [16].

As outlined above, both types of expenditures are regarded as complements in Keynesian tradition. In a relatively earlier study, [17] documents this nexus to the extent that the former responds positively to the changes in the latter for the battery of the countries. Strikingly, size of the government has negative effects on private consumption. In line with the Keynesian absolute income hypothesis, [18] investigated the effect of fiscal policy shocks in terms of rising government expenditure through the changes in productivity and thus, real wage channel on private consumption. In a New Keynesian fashion, the findings assert that rising government outlays tend to increase aggregate productivity and real wages to the extent that marginal utility of consumption becomes more predominant compared to leisure [1]. As a more recent evidence, [2] augment the assumption regarding with the households in New Keynesian framework under dynamic stochastic general equilibrium (DSGE) structure. In addition to non-Ricardian behaving household, it is also assumed that households’ consumption purely equals to their respective labor income. It is documented that private consumption favorably reacts to the expansion in government outlays. In another well-known study, [19] derive the analogous outcome by deploying the structural VAR (SVAR) analysis for the U.S economy. By utilizing the panel cointegration methods, [20] argued as to whether government consumption rule-out private consumption for the OECD economies. The findings revealed that both goods were complements rather than being substitutes. In line with the SVAR tradition, following the study carried out by [19], recent contributions to the empirical literature by [21] and [22] attest that government consumption crowds-in private consumption. However, the calibrated DSGE model by [22] revealed the crowding-out effect of government spending on private consumption [23].

Apart from the afore-mentioned framework under which, New Keynesian structure extensively considered by deploying VAR methods, [3] add to debate by assuming households’ preferences, depending on both public and private spending, whereas households are habit forming under the simplistic RBC structure. By conducting VAR analysis on quarterly data spanning over 1948–2005 for the US economy, the results attest the Keynesian hypothesis. However, [24] documented the favorable reaction of private consumption to the government outlays with an alternative approach by deprecating the assumptions of the RBC and the New Keynesian models. With the inclusion of government outlays as a constituent of the representative household’s utility function, it was reported that increase in government outlays yielded an increase in additional utility of private consumption. Furthermore, robustness of the results was satisfied in a case, in which public and private consumption bundle and labor supply are substitutes. Based on the model developed by [6,24] utilized the autoregressive distributed lag (ARDL) approach for Chinese economy over the period 1985–2013 and supported the validity of Keynesian view. Yet, [25] extends the VAR literature by accounting for the imperfect information and wage rigidities that are associated with the coordination failure among workers. By introducing a baseline DSGE specification, the findings revealed the existence of a beneficial response of private consumption to the public expenditure shocks with higher magnitudes in multiplier.

A further strand of the extant literature includes studies that revealed the validity of the crowding-out effect in congruence with the Classical tradition, which dates back to the pioneering study carried out by [23,26]. The associated complementarity nexus also was derived in the study carried out by [7,9,27–31]. The prevailing negative wealth effect appears to corroborate the notion that public expenditure has a deleterious effect on private consumption. With the developments in panel data techniques, cross-country studies have recently become widespread among the scholars. To this end, [1,13] more recently [4,16] disclosed the presence of substitutability. Beyond these studies, [5] discusses whether the nexus is asymmetric or symmetric. Obtained by conducting ARDL and non-linear ARDL (NARDL) methods, the findings suggest not only the existence for asymmetric long-run relationship but also the existence of substitutability between this pair of variables for Egyptian economy. In a more recent study, [32] analyzed the asymmetric effects of fiscal policy on private economic economies, namely are private consumption and private investment expenditures for a sample of 18 Sub-Saharan African countries. The findings suggest that private consumption reacts negatively to the positive government outlay shocks in times of recession, whereas the reaction of the private consumption during the shock period is still negative in times of expansion and becomes insignificant in forecast horizon periods.

The empirical literature also includes a few study that directly address the EU and individual countries across Europe. In an earlier study, [28] concludes that government outlays have delineator influence on private consumption through which adverse wealth effect for the United Kingdom. By incorporating both structural and Euler equations, [33] aim to reveal whether Ricardian equivalence hypothesis or Keynesian preposition is valid for Spain. The findings suggest the little support for Keynesian theory that opines the complementary between the tandems of aggregate demand. In one of the pioneering study, [34] investigated the influence of government consumption shocks on private consumption under the DSGE model for the Euro zone and manifested the validity of the Keynesian view. [35] strengthen the VAR literature by incorporating the approach developed by [19] for Germany. The findings yield that private consumption response positively to the fiscal shocks that are generated by government spending. Similarly, [36] exert the SVAR analysis for Spain and the findings demonstrate that favorable response of private consumption to the government expenditure shocks diminishes in the long run. For the Spanish economy, [37] considered the breakdown of the public expenditures and private consumption by utilizing the disaggregated annual time-series data ranging from 1970 to 2007. It was reported that barring for the public expenditure on health, the other types of public expenditures tend to influence private consumption in same category positively. [38] who incorporated the quarterly data of the period from 1981 to 2005 for the case of the UK also considered the impact of various categories of public expenditure. The findings verified the validity of negative wealth effect as demonstrated by many scholars in the RBC tradition. Moreover, it was documented that private consumption only reacts positively to the social spending. Apart from these studies, [39], employing a novel approach, analyzed the impact of regional variations in public expenditure on private consumption expenditure of the families that live in various regions of Italy. Except for the public healthcare outlays, spending of the families tends to rise with the increases in various types of public expenditures. Besides these studies that mainly focus on European economies by employing the DSGE model in a New Keynesian framework, the empirical literature include recent contributions by [21,22,40,41], and [42]. Except for [22], crowding-in effect of government purchases was reported in the rest of the studies. Apart from these studies, by utilizing the DSGE model in a New Keynesian framework, [43] reported the validity of the Keynesian predictions. Accordingly, under the presence of nominal price rigidities, private consumption positively reacts to public outlays only if they have productive characteristics (i.e., education, health).

In the light of recent developments in time-series and panel data techniques, many studies verified the validity of either Keynesian predictions or neoclassical predictions regarding the nexus between public spending and private consumption. To this end, [44] examined the presence of either crowding-out or crowding-in effect of government outlays on private consumption by utilizing annual panel data for 145 countries. Their findings revealed the validity of Keynesian predictions to the extent that increasing government outlays tend to crowd-in private consumption. In another study, [45] investigated the impact of government spending on private consumption at a spatial level by incorporating the panel data of Japanese prefectures. After controlling for the spatial autocorrelation effect for private consumption, the findings revealed that increasing government outlays rule-out private consumption [23]. In a recent study, [23] employed a continuous wavelet coherence approach by utilizing a quarterly data set for the Republic of Korea to identify how government consumption influences private economic activities. The findings revealed that government consumption tends to elbow-out private consumption at high frequencies, especially before 1970.

In sum, the findings obtained from the previous studies for European case suggest different results regarding the prevalence of crowding-out or crowding-in effect as long as both theoretical and empirical studies rather than European context. Despite the presence of numerous studies, this paper aims to develop a relatively different approach, which has been sparsely handled in the empirical literature with a few exceptions [36,37] by using macro-level data.

## Model design, data and pre-testing

As outlined in the previous section, the present study aims to unravel the existence of crowding-out or crowding-in effect of government outlays on private consumption by considering the breakdown of the government outlays. In this regard, the present study’s modelling strategy is based on the strategy, which was pioneered by [46] and developed in the empirical studies carried out by [1,4,5]. Besides the consideration of the disaggregated data for government outlays, the present study also differs with the inclusion of the inflation and unemployment in the single model frame that likely induce some fuzziness in private consumption. Accordingly, the following baseline model specifications in log-linear form will be estimated throughout the empirical strategy that will be outlined in the next section:


$$\text{Model }\text{1:}\;{LNPCE}_{it}=\beta_{\text{0}}+\beta_{\text{1}}{LNG\text{1}}_{it}+\beta_{\text{2}}{LNDPI}_{it}+\beta_{\text{3}}{LNCP}_{it}+\beta_{\text{4}}U_{it}+\varepsilon_{it}$$ (1)

$$\text{Model }\text{2:}\;{LNPCE}_{it}=\beta_{\text{0}}+\beta_{\text{1}}{LNG\text{2}}_{it}+\beta_{\text{2}}{LNDPI}_{it}+\beta_{\text{3}}{LNCP}_{it}+\beta_{\text{4}}U_{it}+\varepsilon_{it}$$ (2)

$$\text{Model }\text{3:}\;{LNPCE}_{it}=\beta_{\text{0}}+\beta_{\text{1}}{LNG\text{3}}_{it}+\beta_{\text{2}}{LNDPI}_{it}+\beta_{\text{3}}{LNCP}_{it}+\beta_{\text{4}}U_{it}+\varepsilon_{it}$$ (3)

$$\text{Model }\text{4:}\;{LNPCE}_{it}=\beta_{\text{0}}+\beta_{\text{1}}{LNG\text{4}}_{it}+\beta_{\text{2}}{LNDPI}_{it}+\beta_{\text{3}}{LNCP}_{it}+\beta_{\text{4}}U_{it}+\varepsilon_{it}$$ (4)

In each model, reliant variable is the natural logarithm of private consumption expenditure (LNPCE_it_), whereas LNDPI_it_, LNCP_it_, U_it_ denote the natural logarithm of real disposable income, natural logarithm of consumer price index, and unemployment rate. In each model, ε_it_ denotes the disturbance term, while LNG1_it_, LNG2_it_, LNG3_it,_ and LNG4_it_ represent the various types of government expenditures, which are general government expenditure, defense expenditure, health expenditure, and education expenditure, respectively. Accordingly, the present study aims to test the validity of the following two competing hypotheses respectively:

- H_0_: General government expenditure and distinct categories of government expenditures rule-out private consumption.
- H_a_: General government expenditure and distinct categories of government expenditures stimulate private consumption.

To test the validity of these hypotheses, the present study utilizes the sample of all the EU countries, except for Bulgaria and Romania, which lack suitable data, over the period of 1995−2022. All the data regarding the variables specified above were compiled from the AMECO database of the European Commission’s (EC) Directorate General for Economic and Financial Affairs (DGEFA) annually [47]. Except for unemployment rate, which is the percentage of unemployed persons in the labor force, rest of the variables were converted to their natural logarithmic forms to overcome skewness, heteroscedasticity and stabilize the error variances. Furthermore, it provides a convenient way to interpret the coefficients as their elasticity form, which is economically reasonable. As shown in Table 1, the descriptive statistics and correlation matrix are collated. The mean value of general government expenditure and other types of government expenditures range between 7.400 and 11.066. On the other hand, the mean values of private consumption and disposable income are closely concomitant. The largest standard deviation was observed for the series of unemployment rate, which indicates a strong disparity across countries and periods as the gap between the maximum and minimum values is the largest among all the other variables. Moreover, the results of skewness, kurtosis, and Jarque-Bera tests were examined jointly; it was determined that all the variables are non-normally distributed. Particularly, the non-normality is observed for the series of inflation and unemployment to the extent that skewness, kurtosis and the Jarque-Bera statistics have relatively higher values than the rest of the variables. Most of the economies were negatively influenced during the global financial turmoil and the European debt crisis. As economies have contracted and stagnated, unemployment rates have dramatically surged in some economies. Unlike the unemployment rates, inflation rates tend to decrease in those times. However, the recent surge in both energy and food prices post COVID-19 period has induced in increase in inflation as well. Thus, maximum and minimum values of these variables tailed and lopsided either positively or negatively. Below segment of Table 1 displays the correlation matrix among the variables of interest. Accordingly, disposable income and government outlays are highly and positively correlated with private consumption expenditure.

**Table 1:** Summary statistics and correlation matrix.

A. Summary statistics								
Variables	LNPCE	LNDPI	LNG1	LNG2	LNG3	LNG4	LNCP	U
Observations	700	700	700	700	700	700	700	700
Mean	11.240	11.635	11.066	7.400	9.030	8.898	4.528	8.681
Standard Deviation	1.587	1.611	1.669	1.712	1.746	1.561	0.216	4.374
Minimum	7.822	8.155	7.366	3.599	5.039	5.319	3.358	1.805
Maximum	14.326	14.783	14.274	10.618	12.505	11.857	5.023	27.686
Skewness	0.043	0.001	−0.074	−0.083	−0.137	−0.052	−1.219	1.302
Kurtosis	2.218	2.193	2.211	2.309	2.261	2.130	5.521	4.982
Jarque-Bera	18.024	18.952	18.789	14.708	18.103	22.378	358.96	312.67
Probability	0.000	0.000	0.000	0.000	0.000	0.000	0.000	0.000
**B. Correlation matrix**								
**Variables**	**LNPCE**	**LNDPI**	**LNG1**	**LNG2**	**LNG3**	**LNG4**	**LNCP**	**U**
LNPCE	1.000							
LNDPI	0.997	1.000						
LNG1	0.993	0.9	1.000					
LNG2	0.971	0.967	0.963	1.000				
LNG3	0.984	0.990	0.993	0.945	1.000			
LNG4	0.989	0.994	0.994	0.963	0.988	1.000		
LNCP	0.199	0.213	0.215	0.159	0.253	0.216	1.000	
U	0.044	0.009	0.010	0.064	−0.019	−0.012	−0.198	1.000

Note: The computation of statistics for all variables is conducted in their natural logarithms.

Source: Research findings.

Before focusing on empirical treatment, it is conventional to check for the homogeneity of the slope coefficients and the cross-sectional dependence in baseline models under investigation. Given that the countries in the present sample are constituent members of the European Union, it is empirically tenable to hypothesize that any external shock (commercial, fiscal, economic, etc.) and policy changes may have an influence on them. Correspondingly, under the aegis of cross-sectional dependency (CD), policy shifts or shocks in one state may have a significant impact on the other countries within the sample. To this end, an array of CD tests introduced by [48] were conducted, and the concomitant results with respect to each model specification are shown in the upper portion of Table 2. Upon rejecting the null hypothesis of no CD, the results unequivocally indicate the existence of CD across the sample with respect to each model specification. On the other hand, lower part of Table 2 highlights the results of the homogeneity tests suggested by [49] that are pertaining to each linear model ascription. In this respect, the results of ∆̃ and ∆̃_adj_ tests clearly reject the null hypothesis of the homogeneity of the slope parameters. As such, accounting for the homogeneity constraint on the slope parameters would likely yield inconsistent outcomes for further analyses.

**Table 2:** CD and slope homogeneity tests.

A. CD tests				
	(1)	(2)	(3)	(4)
CD Test	42.491*	43.258*	40.981*	37.988*
CD_LM_ Test	78.455*	83.500*	74.282*	64.943*
LMadj Test	80.746*	92.070*	73.102*	63.669*
**B. Slope homogeneity tests**				
	(1)	(2)	(3)	(4)
Δ Test	22.612*	23.689*	23.199*	21.956*
Δ_adj_ Test	25.509*	26.725*	26.173*	24.770*

Notes: * represents the significance level at 1%.

Under the veneer of CD and heterogeneity, it is also necessary to check for the stationary of the series. Without elimination of the unit root problem, it may lead to the problem of spurious regression as well as the existence of cointegration relationship and the reliability of long-run parameter estimates. Therefore, it is important to examine the degree of integration of the series. For this purpose, the unit root tests developed by [50] (hereafter IPS) and [51] were conducted, and the results are presented in Table 3. Conceived by [50], the ensuing group mean statistics was utilized to test the null hypothesis, which is the series contain unit root in the ambit of slope heterogeneity and normal distribution:

**Table 3:** Panel unit root tests.

	IPS Test			CIPS Test		
Variables	Level	1^st^ Difference	Inference	Level	1^st^ Difference	Inference
LNPCE	−0.886	−13.889*	I(1)	−2.223	−3.986*	I(1)
LNDPI	−1.022	−15.908*	I(1)	−2.477	−4.419*	I(1)
U	−4.025*	−8.781*	I(0)	−2.289	−3.307*	I(1)
LNCP	−0.506	−9.597*	I(1)	−3.129*	−3.763*	I(0)
LNG1	−0.684	−15.181*	I(1)	−2.946*	−5.154*	I(0)
LNG2	0.141	−17.621*	I(1)	−2.616**	−5.289*	I(0)
LNG3	−0.154	−11.811*	I(1)	−2.351	−4.649*	I(1)
LNG4	0.785	−13.122*	I(1)	−2.863*	−4.860*	I(0)

Notes: *,**, and *** represent the significance levels at 1%, 5%, and 10%; the corresponding critical values are −2.81, −2.66, and −2.58, respectively.


$$W_{i}=\frac{\sqrt{N}\left[\overset{―}{t}-\mu_{\overset{―}{t}}\right]}{\sigma_{\overset{―}{t}}}∼N(\text{0},\text{1})$$ (5)

Where $\overset{―}{t}$ denotes the ADF test statistics for each country in the sample. The null hypothesis of the existence of unit root is tested over the alternative hypothesis. However, the principal limitation inherent in first-generation tests pertains to the disregard of CD and heterogeneity considerations in conjunction, which may consequently precipitate erroneous analyses in subsequent phases. To this end, [51] proceeded to develop a unit root test, which was inspired from [50] by incorporating the mean values of lagged levels and taking into cognizance the respective differences for each individual unit, thereby allowing for the CD. In essence, cross-sectionally augmented IPS (CIPS) statistics are derived from cross-sectionally augmented Dickey-Fuller (CADF) statistics through the following representation [52]:


$$CIPS\;\left(N,T\right)=\frac{\text{1}}{N}\sum_{i=\text{1}}^{N}t_{i}\left(N,T\right)$$ (6)

The null hypothesis of the existence of the unit root is tested over the alternative, which is analogous to the one introduced by [50].

The results of unit root tests are shown in Table 3. Accordingly, both tests yielded some significant results. In this respect, the series of unemployment rate (U) is stationary at level with respect to IPS test, whereas it is integrated at I (1) with respect to the CIPS test. However, the opposite applies to the series of inflation. Considering the IPS test results, the series of LNCP becomes stationary by first differencing, while it is stationary at the level, i.e., I (0) according to the test statistics documented by the CIPS test. On the other hand, both tests yielded even more compelling evidence regarding the stationary status of the series for various types of public expenditure. Barring for the series of health expenditure (LNG3), rest of the series are stationary at different integration levels as evidenced by the outcomes of both tests. Finally, both tests for the stationarity of consumption and disposable income series produce consistent results. Despite the various contradicting results that are evidenced by each test, the findings revealed by the CIPS test will be rather considered for further analyses, since it accounts for both CD and slope heterogeneity.

## Estimation approach

Pursuant to the baseline models highlighted above, the empirical scrutiny on whether the various types of public expenditure elbow out private consumption expenditure entails three stages.

### Panel cointegration tests

A rigorous investigation into the long-run association is carried out in a bifurcated approach by conducting the cointegration tests. In this regard, [53] augments the [54] variance ratio type of unit root test by which developing two simple residual-based tests that do not impose correction for temporal dependencies. Additionally, the tests can address not only individual-specific parameters. Therefore, [53] deems the K + 1 dimensional vector of integrated variables of $z_{it}={\left(y_{it},x_{it}^{\prime }\right)}^{\prime }$ which has the following data generating process [53]:


$$z_{it}=z_{it-\text{1}}+v_{it}$$ (7)

Where the vector $v_{it}$ is considered cross-sectionally independent. When the components of the vector of variables are cointegrated, then the residual series become stationary. The variance ratio tests are obtained through the autoregressive parameter of the regression residuals depending on the homogeneity of the parameter. In this regard, the following tests are suggested in examining the cointegration relationship [53]:


$${VR}_{g}=\sum_{i=\text{1}}^{N}\sum_{t=\text{1}}^{T}\hat{E_{it}^{\text{2}}}\hat{R_{i}^{-\text{1}}}\;and\;{VR}_{p}=\sum_{i=\text{1}}^{N}\sum_{t=\text{1}}^{T}\hat{E_{it}^{\text{2}}}{\left(\sum_{i=\text{1}}^{N}{\hat{R}}_{i}\right)}^{-\text{1}}$$ (8)

Where $\hat{E_{it}}\equiv \sum_{j=\text{1}}^{T}\hat{e_{ij}}$ and $\hat{R_{i}}\equiv \sum_{t=\text{1}}^{T}\hat{e_{it}^{\text{2}}}$. The panel statistics (VR_p_) is derived by the aggregation of the distinct terms over each cross-section before multiplication, whereas the group mean statistics (VR_g_) is obtained by multiplying the distinct terms followed by aggregating them over the cross-sectional dimension. Then, the panel test statistics is used in testing for the null hypothesis, i.e., $H_{\text{0}}:p_{i}=\text{1}$ for all i against $H_{\text{1}}:p_{i}=p$, whereas the group mean statistics is used in testing for the null hypothesis, i.e., $H_{\text{0}}:p_{i}=\text{1}$ for all i against $H_{\text{1}}:\left|\rho_{i}\right|<\text{1}$ [53].

Despite producing for the panel and group mean test statistics, as outlined above VR test postulates cross-sectional independency of the integrated terms. In this case, it is also become significant to notice the presence of CD in panel data. Known as second-generation cointegration tests, both CD and heterogeneity is considered. To that end, the present paper also addresses the cointegration relationship across the baseline model specifications by employing DH test pioneered by [55]. Intrinsically, CD is addressed by employing the common factor approach. Notable feature emanates by considering the unit root in the reliant variable, while the independent variable(s) may or may not contain a unit root. Westerlund’s methodology involves the implementation of the Durbin-Hausman test (DHT), in which the lagged difference between the ordinary least squares (OLS) and instrumental variable (IV) estimators of the parameter of interest is instrumented by the first lag of the idiosyncratic error term on itself and thus, obtain the following test statistics respectively [55]:


$${DH}_{p}=\hat{S_{n}}{\left(\tilde{\emptyset }-\hat{\emptyset }\right)}^{\text{2}}\sum_{i=\text{1}}^{n}\sum_{t=\text{2}}^{T}{\hat{e}}_{it-\text{1}}^{\text{2}}\;and\;{DH}_{g}=\sum_{i=\text{1}}^{n}\hat{S_{i}}{\left(\tilde{\emptyset_{i}}-\hat{\emptyset_{i}}\right)}^{\text{2}}\sum_{t=\text{2}}^{T}{\hat{e}}_{it-\text{1}}^{\text{2}}$$ (9)

Where $\hat{\emptyset_{i}}$ and $\hat{\emptyset }$ denote the OLS estiamtors of $\emptyset_{i}$ whereas $\tilde{\emptyset_{i}}$ and $\tilde{\emptyset }$ denote the IV estimators, which can be used in constructing the DHT since the latter is consistent only under the null hypothesis while the former is coherent under both hypotheses.

### Parameter elasticity estimation

By assuming the presence of cointegration relationship among the variables of interest, the next step is to estimate the parameter elasticities with respect to each baseline model specification. For this purpose, the augmented mean group (AMG) estimation technique is to be utilized. [56] introduced a two-step estimator. The prominent benefit of this approach is its efficacy in handling small sample sizes. The incorporation of group-specific fixed effects alongside a set of common factors modelled with country-specific factor loadings would enable the model to accommodate cross-sectional dependency. [56] postulated a series of conditions pertinent to the variables, parameters and error terms. The factor loadings are presumed to have finite sample properties, and the variables are not necessarily subject to a stationary process. It also explains cross-sectional dependency by making use of a common dynamic process (CDP), retrieved by the elimination of the time dummy coefficients of first-differenced standard pooled regression:


$$(i)\Delta y_{it}=b^{\prime }\Delta x_{it}+\sum_{t=\text{2}}^{T}c_{t}D_{t}+e_{it}\to \hat{c_{t}}\equiv \hat{\mu_{t}^{\cdot }}$$ (10)

$$(ii)\;y_{it}=\alpha_{i}+b_{i}^{\prime }x_{it}+c_{i}t+d_{i}\hat{\mu_{t}^{\cdot }}+e_{it}$$ (11)

$$b_{\hat{AMG}}=\frac{\text{1}}{N}\sum_{i=\text{1}}^{N}\hat{b_{i}}$$ (12)

In the first stage of the AMG estimation process, the conventional first-differenced pooled regression model is implemented, incorporating T-1 time dummies, denoted by$\hat{\mu_{t}^{\cdot }}$. In the second stage (ii), time dummy ($\hat{\mu_{t}^{\cdot }}$) is incorporated into each regression. This involves the linear trend term, with a concomitant objective of capturing the influence of the omitted idiosyncratic process, which evolves linearly over time.

### Panel quantile regression method

Both to furnish the robustness and for the sake of benchmarking, the Methods of Moments Quantile regression (MMQR) technique, which was introduced recently by [57], was also conducted in the present study. This method offers more accurate estimation results than OLS estimation methods in case of non-normal distribution and outliers. While the conditional mean is modelled in OLS estimation method, different points of the distribution can be included in the model in quantile estimation methods. Besides this advantage, quantile methods provide results that are more comprehensive. Methods of Moments Quantile regression (MMQR) method provides robust estimates, especially in cases of heterogeneity and non-normal distribution, allowing the effects at different points of the dependent variable to be analyzed. This method entails the inclusion of unobservable fixed effects in the model using the moment approach and the following location-scale model in estimating the conditional quantile regression was formulated:


$$Q_{Y}\left(\tau |X_{it}\right)=\left(\alpha_{i}+\delta_{i}q(\tau )\right)+{X^{\prime }}_{it}\beta +Z_{it}^{\prime }\gamma q(\tau )$$ (13)

Where $Q_{Y}\left(\tau |X_{it}\right)$ represents the conditional value of the dependent variable at quantileτ. Yet, $X_{it}^{\prime }$ represents the vector of explanatory variables, whereas Z_it_ represents the variables that influence the scale of the distribution. In other words, [57] argue that each component of vector Z consists of known in all cases differentiable transformations described of the components of the explanatory variable vector and that the dimension of vector Z has the same dimension as the vector of explanatory variables. Furthermore, in the MMQR model, $\alpha_{i}+\delta_{i}q(\tau )$ represents the fixed effects for each EU country quantile τ fixed effects or the distributional effect at this quantile. Accordingly, the estimation results and the findings will be presented in the next section.

## Findings

### Panel cointegration tests results

The panel variance ratio (VR) and panel Durbin-Hausman (DH) type of cointegration tests were conducted to investigate the long-run interaction among the variables, and the relevant outcomes are presented in Table 4. Panel A presents the results of the VR test, in which group and panel tests were computed separately for the cases of heterogeneity and homogeneity. Even though the results of panel test statistics (VR_p_) cannot reject the null hypothesis of no cointegration with respect to each model specification, the opposite applies to the case of group test statistics (VR_g_). In other words, the computed group test statistics (VR_g_) documents the existence of cointegration relationship. As such, the null hypothesis could be rejected at 10% significance level for all models. Thus, the cointegration relationship tends to exist since private consumption expenditure becomes reliant variable with respect to each baseline model specification to the extent that various public expenditures are involved in. Nevertheless, the main drawback associated with the VR test arises by ignoring the presence of CD. Thus, panel DH cointegration test was conducted, and the lower part of Table 4 discloses the corresponding results. DH test provides two distinct test statistics as long as the existence of CD and slope heterogeneity. Considering the panel test statistics (DH_p_), cointegration relationship tends to exist only for model two and model three. However, group test (DH_g_) statistics are more reliable as long as CD and slope heterogeneity exist in the panel data. In this respect, the computed test statistics are significant at 5% significance level for model 2 and model 4, whereas for model 1 and model 3 are significant at significance levels of 1% and 10%, respectively. This renders the null hypothesis highly implausible across all model specifications. These findings also offer some insights regarding the joint significance of the explanatory variables on private consumption with respect to each baseline model specifications. Given the VR test results, the explanatory variables in model 3 have the strongest effect on private consumption. Along with CD and heterogeneity, the stationary nature of the series at different levels made the DH test more viable. Accordingly, the joint significance of the explanatory variables in model 1, in which general government outlays proxy the government spending, are rather more pronounced compared to the other model specifications.

**Table 4:** Panel cointegration tests.

A. Panel VR cointegration test				
Test Statistics	(1)	(2)	(3)	(4)
VR_p_	−0.402(0.343)	−0.861(0.194)	−0.769(0.220)	−0.209(0.416)
VR_g_	−1.389(0.082)***	−1.432(0.076)***	−1.541(0.061)***	−1.258(0.100)***
**B. Panel DH cointegration test**				
Test Statistics	(1)	(2)	(3)	(4)
DH_p_	−0.856(0.196)	−1.294(0.098)***	−1.914(0.028)**	0.708(0.760)
DH_g_	−2.566(0.005)*	−2.141(0.016)**	−1.453(0.073)***	−1.709(0.044)**

Notes: *, **, and *** represent the significance levels at 1%, 5%, and 10%, respectively. For both tests, p-values are shown in parentheses.

### Long-run elasticity results

Under the condition of cointegration relationship, the next stage aims to reveal the direction and magnitude of the parameter elasticities. Accordingly, the AMG estimator was utilized, and the relevant results are presented in Table 5. It is clearly shows that general government outlay and private consumption are positively interacted. As such, one percent rise in general government expenditure upswings private consumption by 0.20 percent. Hence, general government outlays tend to crowd-in private consumption. This finding confirms the validity of Keynesian notion, which postulates that rising government outlay induces private consumption to increase via multiplier effect to the extent that output and thus disposable income surges. Furthermore, the results also indicate that disposable income has conducive influence on private consumption. In this context, one percent raise in disposable income increases private consumption by 0.53 percent, which indicates the validity of Keynesian consumption theory. On the other hand, private consumption is negatively related with two major macroeconomic aggregates in the long run. To this end, a point increase in unemployment rate, which is the indication of the uncertainty associated with income, decreases private consumption by 0.25. However, the negative effect of inflation on private consumption is more profound to the extent that rising inflation tends to reduce private consumption by decreasing the purchasing power of the households. In this respect, one percent rise in inflation rate reduces the private consumption by 0.18 percent.

**Table 5:** Augmented mean group estimation results.

A. General government outlays				
**Dependent variable: LNPCE**				
Variables	LNDPI	U	LNCP	LNG1
	0.5338(0.0496)*	−0.0025(0.0014)***	−0.1865(0.0491)*	0.1957(0.0374)*
RMSE = 0.0186				
Wald χ^2^ = 530.85[0.0000]*				
Observations = 700				
**B. Defense expenditure**				
**Dependent variable: LNPCE**				
Variables	LNDPI	U	LNCP	LNG2
	0.6594(0.0487)*	−0.0004(0.0018)	−0.0798(0.0680)	−0.0068(0.0155)
RMSE = 0.0196				
Wald χ^2^ = 404.44[0.0000]*				
Observations = 700				
**C. Health expenditure**				
**Dependent variable: LNPCE**				
Variables	LNDPI	U	LNCP	LNG3
	0.5513(0.0490)*	−0.0016(0.0013)	−0.1637(0.0530)*	0.1125(0.0292)*
RMSE = 0.0191				
Wald χ^2^ = 407.89[0.0000]*				
Observations = 700				
**D. Education expenditure**				
**Dependent variable: LNPCE**				
Variables	LNDPI	U	LNCP	LNG4
	0.5323(0.0430)*	−0.0006(0.0015)	−0.1272(0.0637)**	0.1765(0.0302)*
RMSE = 0.0188				
Wald χ^2^ = 611.11[0.0000]*				
Observations = 700				

Notes: *, **, and *** represent the significance levels at 1%, 5%, and 10% respectively. The standard errors are shown in parentheses, whereas p-values of Wald test statistics are shown in the brackets.

Table 5 also reports the results with respect to breakdown of the government outlays. Except for defense expenditure, rest of the government expenditures are positively interacted with private consumption. In this respect, one percent rise in health expenditure tends to accelerate private consumption by 0.11 percent whereas one percent rise in education expenditure tends to accelerate private consumption by almost 0.18 percent. Thereby, the validity of the Keynesian notion is indicated in the context of two types of public expenditure. Moreover, the results reveals the presence of Absolute Income Hypothesis positive with respect to each specification in which various types of government outlays were considered as the proxy for government spending. Nonetheless, the magnitude of the coefficient for disposable income varies by each specification.

### Panel quantile regression results

To ensure the robustness of the previous results and since the variables are non-normally distributed, the quantile regression technique was employed, and the related estimation results are presented in Table 6. The estimation results of MMQR suggest that the location coefficient of general government expenditure is positive, which accentuates that private consumption is fueled by the increase in general government expenditure. Similarly, disposable income has an accelerating effect on private consumption since the location coefficient is positive and statistically significant. It should also be indicated that the location coefficients of inflation and unemployment are insignificant. On the other hand, general government outlay tends to have an expansionary effect on the distribution of private consumption because the scale coefficient is positive and statistically significant. However, the distribution of private consumption is negatively influenced by disposable income and inflation because the scale coefficients of both variables are negative. Yet, unemployment has no significant effect on the distribution of private consumption. These results also suggest that public outlay tends to crowd-in private consumption expenditure. This effect is statistically significant in all quantiles, except for the 0.10 quantile. It is determined that the effect of general public outlay grows as it is moved from low to high quantiles. Accordingly, whilst a 1% increase in general public outlay in quantile 0.25 increases private consumption expenditure by 0.1398%, it boosts private consumption expenditure by 0.2014%, 0.2570%, and 0.2929% in quantiles 0.50, 0.75, and 0.90, respectively. Given the results of Model 1, it was also determined that the real disposable income (LNDPI) positively influences private consumption expenditure in each quantile even though the value of its coefficients tends to diminish as it goes to higher quantiles. The effects of unemployment rate are more pronounced at quantiles 0.10, 0.25, and 0.90. However, the effect is positive at quantiles 0.10 and 0.25, but this positive effect is so slight and can be considered insignificant. Yet, inflation rate (LNCP) does not have any significant effect on reliant variable in all quantiles.

**Table 6:** Panel quantile regression results.

Model 1				Quantiles			
Variables	Location	Scale	0.10	0.25	0.50	0.75	0.90
LNG1	0.1953***	0.0651**	0.0905	0.1398***	0.2014***	0.2570***	0.2929***
LNDPI	0.6967***	−0.0732**	0.8147***	0.7591***	0.6899***	0.6273***	0.5869***
U	0.0010	−0.0016**	0.0036**	0.0024*	0.0009	−0.0004	−0.0013*
LNCP	0.0043	−0.0099	0.0204	0.0128	0.0033	−0.0051	−0.0106
**Model 2**				**Quantiles**			
LNG2	0.0107	−0.0026	0.0148	0.0130	0.0106	0.0083	0.0066
LNDPI	0.8763***	−0.0088	0.8900***	0.8837***	0.8757***	0.8681***	0.8625***
U	0.0034***	−0.0008**	0.0048***	0.0042***	0.0034***	0.0026***	0.0021***
LNCP	0.0257*	−0.0066	0.0361*	0.0314*	0.0253*	0.0196	0.0153
**Model 3**				**Quantiles**			
LNG3	0.0778***	0.0301***	0.0310	0.0520**	0.0788***	0.1062***	0.1224***
LNDPI	0.7954***	−0.0462***	0.8674***	0.8351***	0.7938***	0.7517***	0.7269***
U	0.0029***	−0.0011***	0.0047***	0.0039***	0.0028***	0.0018***	0.0012*
LNCP	0.0047	−0.0087	0.0183	0.0122	0.0044	−0.0035	−0.0082
**Model 4**				**Quantiles**			
LNG4	0.1757***	0.0314**	0.1280***	0.1474***	0.1773***	0.2057***	0.2224***
LNDPI	0.7063***	−0.0380***	0.7641***	0.7406***	0.7044***	0.6701***	0.6497***
U	0.0017***	−0.0009***	0.0032***	0.0026***	0.0017***	0.0008	0.0004
LNCP	0.0282**	−0.0068	0.0385**	0.0343**	0.0279**	0.0217*	0.0181

Notes: *, **, and *** represent the significance levels at 10%, 5%, and 1%, respectively.

When defense expenditure is modelled, the MMQR estimation results indicate that defense expenditure has no significant effect. On the other hand, the effects of LNDPI and U are positive in all quantiles. The effect of the LNCP was found to be positive and significant in the quantiles 0.10, 0.25, and 0.50; however, the effect diminishes in higher quantiles, i.e., 0.75 and 0.90. It should also be highlighted that the location coefficient of the defense expenditure was found be insignificant. However, the location coefficients of the other independent variables are positive and statistically significant to the extent that private consumption is fostered by the increase in these variables. On the other hand, the scale coefficients of the variables do not follow a sporadic trend. Except for unemployment rate, the scale coefficients of the other variables do not have paramount effect on private consumption. Additionally, rises in unemployment rate tend to reduce the distribution of private consumption.

In the case where health expenditure proxy the public expenditure, the influence of health expenditure is positive in all quantiles, except for the quantile 0.10. While a 1% increase in health expenditure in the 0.25^th^ quantile stimulates private consumption expenditure by 0.052%, it increases by 0.0788%, 0.1062%, and 0.1224% in the higher quantiles, respectively. Meanwhile, the effects of LNDPI and U are positive in all quantiles, and LNCP was found to have no significant influence on reliant variable. These results also indicate that the location coefficients of health expenditure, together with disposable income and unemployment, are positive and statistically significant, whereas the location coefficient of inflation rate is insignificant. Moreover, the scale coefficients of the variables do not exhibit uniform tendency. Therefore, rises in LNDPI and U tends to diminish the distribution of private consumption, while the distribution of private consumption expands with the rises in LNG3. Yet, the scale coefficient of LNCP was determined to be insignificant.

Besides, the results of the model, in which education expenditure is chosen as the proxy of public expenditure, indicate that an increase in education expenditure fuels the rise in private consumption expenditure for all quantiles. Accordingly, a 1% rise in LNG4 leads to an increase of 0.1280%, 0.1474%, 0.1773%, 0.2057%, and 0.2224% for all quantiles, respectively. Concurrently, it was observed that real disposable income has a positive effect on private consumption expenditure in all quantiles, corroborating the Keynesian absolute income hypothesis. The increase in unemployment rate has an accelerating effect on private consumption expenditure for quantiles 0.10, 0.25, and 0.50. Finally, whilst inflation has a positive effect on private consumption expenditure for all quantiles, it is significant for all quantiles, except for quantile 0.90. Unlike the other model specifications, the location coefficients of variables are positive, indicating that rises in independent variables tend to accelerate the reliant variable.

**Fig 6:**
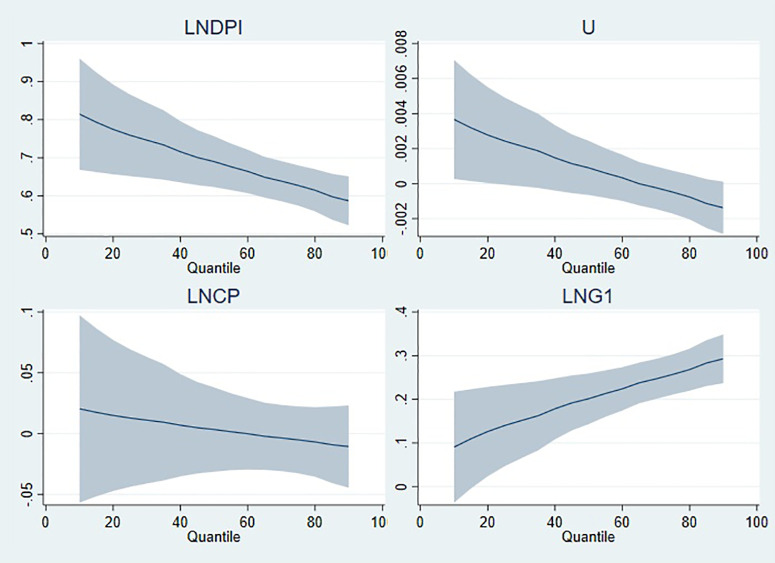
Quantile illustration of the coefficients in model 1.

**Fig 7:**
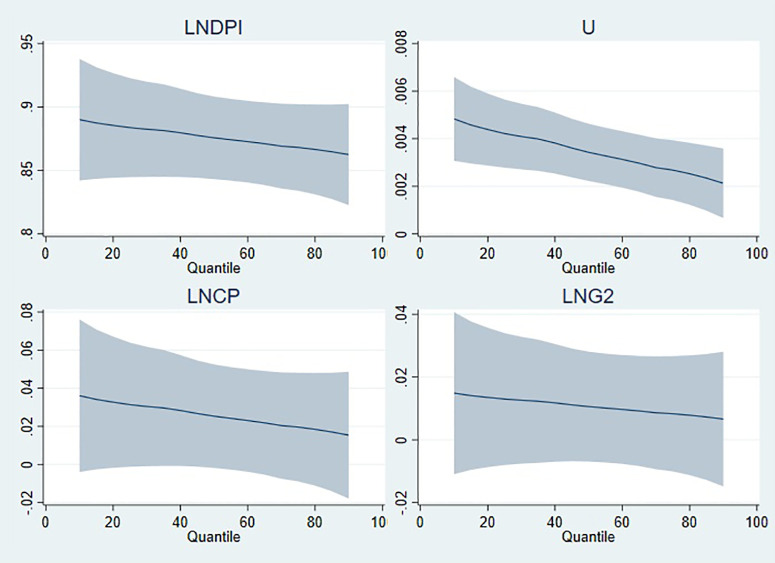
Quantile illustration of the coefficients in model 2.

**Fig 8:**
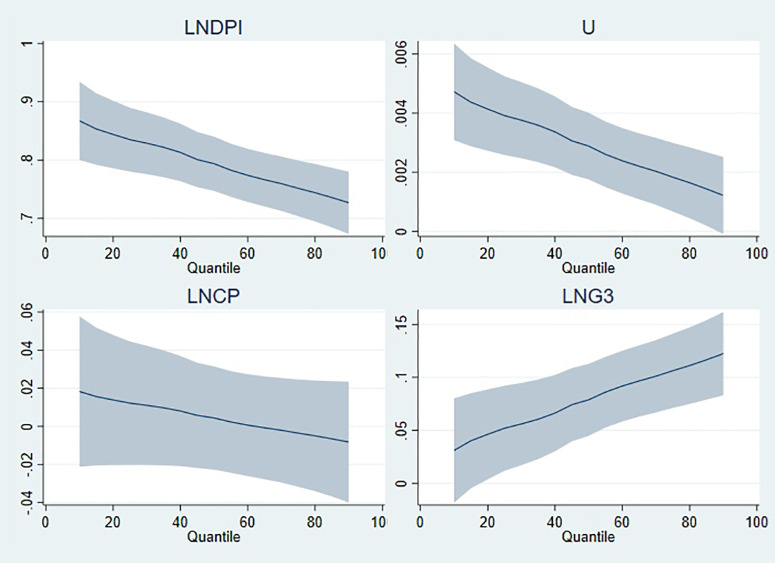
Quantile illustration of the coefficients in model 3.

**Fig 9:**
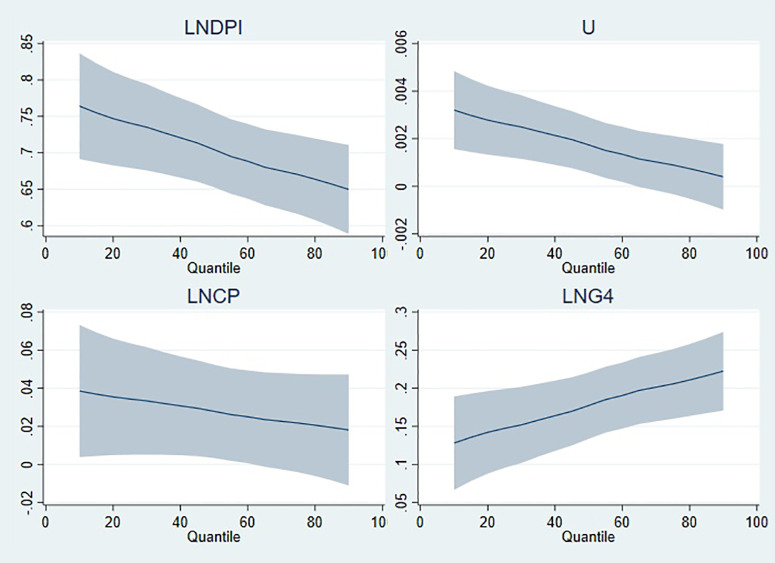
Quantile illustration of the coefficients in model 4.

On the other hand, Figs 6–9 illustrate the graphical representation of the coefficients by each model, and it can be clearly seen that the stimulating effects of public expenditure on private consumption expenditure manifest itself at high quantiles. Real disposable income, which has a positive effect on private consumption expenditure, has a tapering effect from low to high quantiles in all models. For the estimation results of model 1 in Fig 6, the positive effect of unemployment gradually fades as the quantile level increases, whereas its negative effect tends to accelerate. In the graphical illustrations in Figs 7–9, it is shown that the positive effect of unemployment decreases as the quantile level increases. Evaluating the graphical illustrations together for all models, it can be seen that the effect of inflation, the coefficients of which are significant for model 2 and model 4, tend to decrease as the quantile level increases. The scale coefficient of LNG4 is positive, suggesting that the distribution of private consumption increases in parallel with the education expenditure (LNG4). However, the scale coefficients of disposable income and unemployment rate have contradictory effects on the distribution of private consumption, whereas inflation does not have significant influence on the distribution as long as its coefficient is insignificant.

## Discussion

In overall, the foregoing findings indicate that public and private consumption expenditure are rather complements as the coefficients of general government outlays and other types of government outlay are positive in AMG and panel quantile regression, except for defense expenditure. Although the surge in defense expenditure may lead to a rise in aggregate demand and hence disposable income through the expansion in public expenditure, it fails to spur private consumption, such as education and health expenditure, which account for a sizeable proportion of individuals’ budgets. Thus, the Keynesian multiplier mechanism cannot function effectively when defense spending is taken as a proxy variable. The studies carried out by [10] and [11] revealed that military spending-rather than non-military spending-negatively affects private consumption. It likely occurs when governments tend to reduce non-military outlays and/or increase taxes, which may have sizeable influences on households’ budgets. Accordingly, the present study lends support to the Keynesian hypothesis, a conclusion that is further reinforced by the findings of previous studies by [1,2,17–19]. However, similar findings were also revealed by [3,24,34,35,37,40,41] and more recently by [6,21,22,25,42,43].

Considering the previous discussions, the findings reveal some compelling evidence in connection with the EU’s Stability and Growth Pact, which was established in 1997. This Pact was amended during the Eurozone crisis in 2010’s and suspended with the outbreak of COVID-19 pandemic in 2020 and the warfare between Ukraine and Russia, aiming to prevent the global demand to slowdown [58]. Yet, a new reform on the Pact came into effect on 29 April 2024 and was approved by the Economic and Financial Affairs Council (ECOFIN), the European Commission, and the European Parliament [59]. The key reference thresholds regarding the government debt to GDP ratio and the government budget deficit to GDP ratio, which are 60% and 3%, respectively, remain same in the reformed version of the Pact [59]. In order to monitor the position of these reference thresholds and fiscal rules associated with the Pact, the reformed version of the Pact introduced net expenditure rule, which consists of primary public expenditure of general government, less interest payments, and less cyclical unemployment benefits [58]. Moreover, the reformed Pact requires the member states to submit their national medium-term fiscal-structural plans, which must be based on the priorities of the EU including a fair green and digital transition, social and economic resilience, energy security, and defense.

On the other hand, main novelties of the reformed Pact are associated with reducing the reference threshold values regarding the debt-to GDP ratio and deficit-to GDP ratio. In this respect, two safeguards were developed. The first safeguard is known as debt sustainability safeguard, which will be applied government deficit fall below the 3% threshold level to the extent that the member states, which have 90% and more debt-to GDP ratio would commit to decreasing by 1% per annum. In addition, the member states, which have debt-to GDP ratio between 60% and 90% would commit to decreasing by 0.5% per annum [58]. The second safeguard is called deficit resilience safeguard, which reinforces the member states to preserve their structural deficit at or below 1.5% of the respective GDP. If any member state exceeds this threshold level, then structural primary deficit should be reduced to 0.4% of GDP per annum [58]. In order to reach these goals, the member states need either to cut public outlays or increase taxes. However, this kind of austerity measures could be detrimental for the growth projections, and hence for private consumption, which is one of the most essential elements of the aggregate demand. Moreover, the priorities of the EU spotlight the importance of the defense expenditure, which has no significant influence or even tend to rule-out private consumption.

In line with the multitude of the foregoing studies, the findings provide substantial prima facie evidence in favor of the Keynesian Absolute Income Hypothesis. Both long time horizon parameter estimates and quantile regression results lend support Keynesian view. Thus, disposable personal income stands out as the most substantial driver of private consumption in the EU countries. As the economies grow faster, disposable personal income and private consumption increase so that the Keynesian multiplier mechanism operates efficiently.

Interestingly, the findings regarding the impact of unemployment on private consumption yield some ambiguities. According to the AMG method, unlike the case in the first model, unemployment rate does not have any significant influence on private consumption in the models when various types of government outlays are involved as explanatory variables. It is can be stated that unemployment has no significant impact on private consumption since it is closely related with the uncertainty regarding the income of the consumers. Rather than military expenditure, non-military expenditures such as education and health are important goods that consumers essentially allocate their limited budget. Thus, rises in unemployment engender the shrinkage in income level, which likely to retard the demand for goods and services.

The long run parameter elasticity estimates reveal that inflation rate is negatively associated with private consumption overall, barring the case in which defense outlays are considered as the proxy for government expenditure. The negative association between the inflation rate and private consumption might be evaluated in two ways. The first mechanism is that inflation induces a shrinkage in private consumption through the well-known phenomenon of money illusion, in which increasing in income attached to the increase in overall price level in the economy stimulate the savings rather than consumption. In this case, consumers perceive the income increase as the real increase rather than nominal and thus, they react as if their well-being is relatively improved. Therefore, they allocate a larger proportion of their income to the savings instead of consumption. Another possible mechanism is related with the financial assets of consumers. By depreciating the real value of financial assets, inflation causes individuals’ wealth to erode, leading to a fall in consumption expenditure.

The findings of quantile regression method revealed some compelling evidence regarding the impact of inflation on private consumption. In contrast to the findings obtained from the AMG method, inflation rate has positive influence on private consumption in models 2 and 4. As the consumers perceive the rises in price level, they tend to consume more of their income as if their wealth would be worsened. Another possible explanation is associated with the expectations. In times of high inflation or volatilities associated with inflation, consumers’ anticipation on prices tend to rise, which in turn leads private consumption to spur. Considering these discussions on the findings, relevant policy recommendations will be developed in the conclusion part of the present study.

## Conclusion and policy recommendations

The change in private consumption expenditure, which is an important component of aggregate demand, can provide important inferences about the overall performance of the economy and the course of the business cycle. Moreover, direction of the changes in private consumption could be influenced by the changes in government expenditure, and this subject has been widely discussed both empirically and theoretically. Despite the presence of extensive ongoing debate, there has not been any consensus regarding the direction of the interaction between this pair of components in aggregate demand. The present study aims to shed light on this interaction by employing recent panel data methods distinctively since many empirical studies focused on the VAR methodology. Given the strong evidence in favor of cointegration relationship with respect to each baseline model specification, it can be stated that general government expenditure tends to crowd-in private consumption expenditure. Except for defense expenditure, the other types of public expenditure are also positively related with private consumption as well, which is also driven by the panel quantile regression method. Overall, Keynesian argument of crowding-in hypothesis holds true for the EU countries.

These findings can yield some useful policy recommendation and directions for the future studies in this field. The complementary nature of public expenditure to consumption expenditure or the existence of a positive inter-connection between them indicates that public expenditure can be effective in fueling aggregate demand as a fiscal policy instrument. Since there is an anticipated interaction between disposable income and private consumption expenditure, expansionary fiscal policy implemented by increasing public expenditures can be effective in stimulating private consumption expenditures. Accordingly, governments can boost aggregate demand by increasing public expenditure in recessionary periods without ruling out private consumption expenditure. However, defense expenditure stands out as an exception. In this respect, the fact that defense expenditure does not have a positive effect on private consumption expenditure, unlike the other categories of public expenditure, indicates that the expansionary fiscal policy increasing defense expenditures may not have the anticipated effect on aggregate demand and private consumption.

The rationale behind the foregoing arguments is the fact that education and health expenditure categories constitute a much larger share of household spending budgets than defense. Thus, these categories have more consumption-enhancing effects than defense. On the other hand, if governments and policymakers decide to increase defense expenditures (as prioritized in the current reformed version of the Stability and Growth Pact), a trade-off between other components of public spending would likely emerge and negatively influence the consumption patterns of the households. Another option for policymakers in financing the defense expenditures would be increasing the taxes. In this case, disposable income of the households might shrink to the extent that tax levied on income particularly, which might adversely influence private consumption.

On the other hand, policymakers should account-consider the budgetary balances, which are subject to some binding rules and measure for the member states and put into effect by the Treaty of Maastricht and Stability and Growth Pact. Therefore, limited budgetary sources should be allocated by considering the budgetary balances and priorities of the EU. Another compelling task for the policymakers is to deal with the EU-wide secular stagnation. Despite the potential beneficial effects on fiscal sustainability, prolonged fiscal austerity measures could be detrimental for economic recovery and aggregate demand. As outlined above, increasing taxes likely affect aggregate demand through private consumption.

The present study also has some limitations. In particular, the lack of a suitable data set to cover all EU countries draws attention as the most important limitation. Besides this substantial limitation, the findings achieved in the present study may shed light on future studies that may be augmented to reveal the influence of distinct categories of public expenditure on various types of private consumption expenditure. Additionally, by incorporating micro or regional level data sets, spatial spillover effects of public expenditure on private consumption can also be observed by the implementation of spatial econometrics analyses.

## Supporting information

S1 Appendix Appendix Tables. (DOCX)

S1 Data Data set. (XLSX)
