# Supplementary material for: Do government outlays crowd-out private consumption? Evidence from the European Union
Source: PLoS One. 2026 Jan 2;21(1):e0336229. doi: 10.1371/journal.pone.0336229 (PMC12758785; doi:10.1371/journal.pone.0336229)
Supplement: S1 Appendix — (DOCX) [file pone.0336229.s001.docx]

**APPENDICES**

**Table A1.** **List of sample countries and abbreviations.**

| **Country Name** | **Abbreviation** | **Country Name** | **Abbreviation** | **Country Name** | **Abbreviation** |
| --- | --- | --- | --- | --- | --- |
| Austria | AUS | Germany | GER | The Netherlands | NED |
| Belgium | BEL | Greece | GRE | Poland | POL |
| Croatia | CRO | Hungary | HUN | Portugal | POR |
| Cyprus | CYP | Ireland | IRE | Slovakia | SLK |
| Czechia | CZE | Italy | ITA | Slovenia | SLV |
| Denmark | DEN | Latvia | LAT | Spain | SPA |
| Estonia | EST | Lithuania | LIT | Sweden | SWE |
| Finland | FIN | Luxembourg | LUX |  |  |
| France | FRA | Malta | MAL |  |  |

**Table A2. List of variables and data sources.**

| **Variables** | **Long Definition** | **Data Source** | **Expected Signs** |
| --- | --- | --- | --- |
| LNPCE | Natural Logarithm of Real | AMECO, EC | Dependent Variable |
|  | Private Consumption Expenditure |  |  |
| LNDPI | Natural Logarithm of Real Disposable Income | AMECO, EC | +, - |
| LNG1 | Natural Logarithm of Real | AMECO, EC | +, - |
|  | General Government Expenditure |  |  |
| LNG2 | Natural Logarithm of Real | AMECO, EC | +, - |
|  | Government Defense Expenditure |  |  |
| LNG3 | Natural Logarithm of Real | AMECO, EC | +, - |
|  | Government Health Expenditure |  |  |
| LNG4 | Natural Logarithm of Real | AMECO, EC | +, - |
|  | Government Education Expenditure |  |  |
| LNCP | Natural Logarithm of Consumer Price Index (2009=100) | AMECO, EC | - |
| U | Unemployment Rate (%) | AMECO, EC | - |
